# Alcohol Use Disorder Treatment Availability at Mental Health Treatment Facilities

**DOI:** 10.1001/jamanetworkopen.2025.20409

**Published:** 2025-07-14

**Authors:** Susan H. Busch, David A. Fiellin, Kaede Iida, Kim Gannon, Melissa B. Weimer, Jason Hockenberry

**Affiliations:** 1 Yale School of Public Health, New Haven, Connecticut; 2 Yale School of Medicine, New Haven, Connecticut; 3 Tobin Center for Economic Policy, Yale University, New Haven, Connecticut

## Abstract

This cross-sectional study assesses whether specialty mental health facilities offer treatment for alcohol use disorder to patients with co-occurring mental health disorders and alcohol use disorder.

## Introduction

Among adults with mental illness, 18.9% or 11 million individuals have co-occurring alcohol use disorder (AUD).^1^ Yet, only 8% of individuals with AUD receive treatment, and less than 2% receive medications for alcohol use disorder (MAUD).^1^ MAUD in particular has been shown to decrease drinking and associated harms from alcohol use.^2^ Among those with co-occurring disorders, remission from both conditions is more likely if each condition is treated.^3^ One approach to increase treatment access is to integrate alcohol treatments into behavioral health care settings. Our objective is to assess whether specialty mental health facilities offer treatment for AUD to patients with co-occurring mental health disorders and AUD.

## Methods

In this cross-sectional study, we used the 2023 National Substance Use and Mental Health Services Survey, an annual survey of all mental health and substance use disorder (SUD) facilities in the US.^4^ We omitted facilities that did not provide outpatient treatment and stratified our sample by whether facilities are primarily mental health facilities (only serving primary mental health and co-occurring SUD) vs those that offer both mental health and SUD treatment as primary services.

We created binary variables indicating whether facilities report providing several SUD services, including MAUD. Facility characteristics included setting, insurance accepted, and accreditation. To proxy for potential MAUD prescriber presence, we examined whether each facility was a federally qualified health center, provided integrated primary care, or provided antipsychotic medication. We estimated the association of facility characteristics and SUD treatment offerings to patients with co-occurring mental health and AUD in bivariate models; Sensitivity analysis considered results in adjusted models with state fixed effects. This study followed the Strengthening the Reporting of Observational Studies in Epidemiology (STROBE) reporting guideline and was deemed not human participant research by the Yale University institutional review board. Data were analyzed using R version 4.4.1 (R Project for Statistical Computing) from August 2024 to December 2024. Statistical significance was set at *P* < .001, and all tests were 2-sided.

## Results

Of 6572 facilities in our analytic sample, 2640 primarily provided mental health services (40.2%) and 3932 offered both mental health and SUD treatment as primary services (59.8%) (Table). The former often provided SUD treatment (1942 [73.6%]), yet only 262 facilities (9.9%) offered MAUD. Among the latter, 1633 facilities (41.5%) offered MAUD. Whether facilities offered SUD treatment and MAUD differed by setting and region. Facilities that accepted insurance, and those that met our proxy condition for having a prescribing clinician were more likely to offer MAUD. Differences by facility characteristics were generally qualitatively similar but attenuated in multivariable models (available from authors upon request). SUD counseling and case management were more likely to be offered than MAUD (Figure).

**Table.** Characteristics of Mental Health Treatment Facilities and Provision of Alcohol Use Disorder (AUD) Treatment Services

Characteristic	Primarily mental health facilities^a^							Facilities that offer both mental health and SUD treatment as primary services^a^			
	Total, No. (column %)	Offers SUD treatment, No. (row %)			Offers MAUD, No. (row %)			Total, No. (column %)	Offers MAUD, No. (row %)		
		No	Yes	*P* value^b^	No	Yes	*P* value^b^		No	Yes	*P* value^b^
Total	2640 (100)	698 (26.4)	1942 (73.6)		2378 (90.1)	262 (9.9)		3932 (100)	2299 (58.5)	1633 (41.5)	
Setting											
Outpatient mental health facility	1756 (66.5)	518 (29.5)	1238 (70.5)	<.001	1603 (91.3)	153 (8.7)	<.001	2189 (55.7)	1351 (61.7)	838 (38.3)	<.001
CMHC	605 (22.9)	122 (20.2)	483 (79.8)		536 (88.6)	69 (11.4)		1046 (26.6)	597 (57.1)	449 (42.9)	
CCBHC	132 (5)	21 (15.9)	111 (84.1)		104 (78.8)	28 (21.2)		487 (12.4)	250 (51.3)	237 (48.7)	
Partial hospitalization/day treatment	147 (5.6)	37 (25.2)	110 (74.8)		135 (91.8)	12 (8.2)		210 (5.3)	101 (48.1)	109 (51.9)	
Census region											
Northeast	648 (24.5)	171 (26.4)	477 (73.6)	<.001	568 (87.7)	80 (12.3)	<.001	585 (14.9)	256 (43.8)	329 (56.2)	<.001
Midwest	651 (24.7)	212 (32.6)	439 (67.4)		565 (86.8)	86 (13.2)		1144 (29.1)	674 (58.9)	470 (41.1)	
South	676 (25.6)	187 (27.7)	489 (72.3)		633 (93.6)	43 (6.4)		931 (23.7)	589 (63.3)	342 (36.7)	
West	665 (25.2)	128 (19.2)	537 (80.8)		612 (92)	53 (8)		1272 (32.3)	780 (61.3)	492 (38.7)	
Insurance											
Medicaid											
Not accepted	229 (8.7)	87 (38)	142 (62)	<.001	223 (97.4)	6 (2.6)	<.001	293 (7.5)	205 (70)	88 (30)	<.001
Accepted	2411 (91.3)	611 (25.3)	1800 (74.7)		2155 (89.4)	256 (10.6)		3639 (92.5)	2094 (57.5)	1545 (42.5)	
Medicare											
Not accepted	965 (36.6)	340 (35.2)	625 (64.8)	<.001	943 (97.7)	22 (2.3)	<.001	808 (20.5)	602 (74.5)	206 (25.5)	<.001
Accepted	1675 (63.4)	358 (21.4)	1317 (78.6)		1435 (85.7)	240 (14.3)		3124 (79.5)	1697 (54.3)	1427 (45.7)	
Private insurance											
Not accepted	582 (22)	172 (29.6)	410 (70.4)	.06	554 (95.2)	28 (4.8)	<.001	388 (9.9)	286 (73.7)	102 (26.3)	<.001
Accepted	2058 (78)	526 (25.6)	1532 (74.4)		1824 (88.6)	234 (11.4)		3544 (90.1)	2013 (56.8)	1531 (43.2)	
Accreditation											
State SU agency											
No	2292 (86.8)	644 (28.1)	1648 (71.9)	<.001	2107 (91.9)	185 (8.1)	<.001	1424 (36.2)	872 (61.2)	552 (38.8)	.009
Yes	348 (13.2)	54 (15.5)	294 (84.5)		271 (77.9)	77 (22.1)		2508 (63.8)	1427 (56.9)	1081 (43.1)	
Joint commission											
No	2004 (75.9)	541 (27)	1463 (73)	.27	1808 (90.2)	196 (9.8)	.72	3045 (77.4)	1825 (59.9)	1220 (40.1)	.001
Yes	636 (24.1)	157 (24.7)	479 (75.3)		570 (89.6)	66 (10.4)		887 (22.6)	474 (53.4)	413 (46.6)	
CARF											
No	2112 (80)	586 (27.7)	1526 (72.3)	.003	1891 (89.5)	221 (10.5)	.08	2699 (68.6)	1563 (57.9)	1136 (42.1)	.31
Yes	528 (20)	112 (21.2)	416 (78.8)		487 (92.2)	41 (7.8)		1233 (31.4)	736 (59.7)	497 (40.3)	
Complementary services offerings											
FQHC											
No	2428 (92)	654 (26.9)	1774 (73.1)	.13	2202 (90.7)	226 (9.3)	<.001	3428 (87.2)	2038 (59.5)	1390 (40.5)	<.001
Yes	212 (8)	44 (20.8)	168 (79.2)		176 (83)	36 (17)		504 (12.8)	261 (51.8)	243 (48.2)	
Integrated primary care											
No	2262 (85.7)	636 (28.1)	1626 (71.9)	<.001	2088 (92.3)	174 (7.7)	<.001	2740 (69.7)	1748 (63.8)	992 (36.2)	<.001
Yes	378 (14.3)	62 (16.4)	316 (83.6)		290 (76.7)	88 (23.3)		1192 (30.3)	551 (46.2)	641 (53.8)	
Reports prescribing of antipsychotic medication											
No	1140 (43.2)	415 (37.3)	715 (62.7)	<.001	1115 (97.8)	25 (2.2)	<.001	1281 (32.6)	1044 (81.5)	237 (18.5)	<.001
Yes	1500 (56.8)	273 (18.2)	1227 (81.8)		1263 (84.2)	237 (15.8)		2651 (67.4)	1255 (47.3)	1396 (52.7)	

Abbreviations: CARF, Commission on Accreditation of Rehabilitation Facilities; CCBHC, certified community behavioral health center; CMHC, community mental health center; FQHC, federally qualified health center; MAUD, medication for alcohol use disorder; SUD, substance use disorder.

^a^ Primarily mental health facilities only offer substance use treatment as part of mental health treatment services for individual patients who need it. Facilities that offer both mental health and SUD treatment as primary services offer SUD services as a stand-alone service.

^b^ χ^2^ tests were used to compare facility characteristics and provision of AUD treatment. *P* values represent 2-sided tests.

The 2023 dataset^4^ included responses from 29 113 facilities and had an 84.9% response rate with over 98% of facilities completing the online survey. We omitted hospitals and residential-only treatment facilities, facilities that only serve individuals seeking treatments for driving under the influence or driving while intoxicated; facilities operated by the Department of Veterans Affairs, Department of Defense, or Indian Health Services; and facilities in Puerto Rico or other jurisdictions due to concerns related to including care provided by federal agencies or more specialized facilities.

**Figure.**
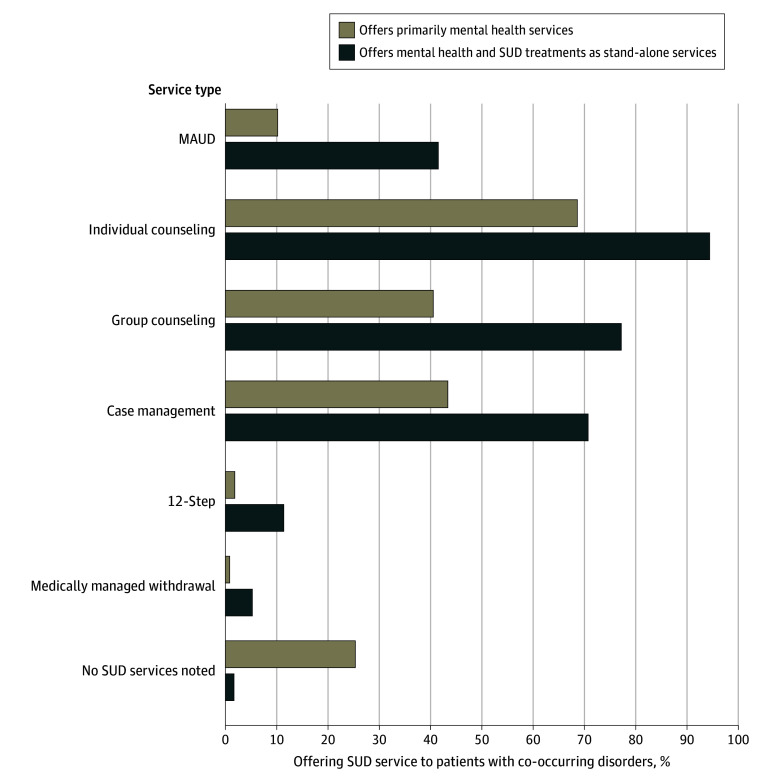
Percentage of Facilities Offering Substance Use Disorder Treatment Services to Patients With Co-Occurring Disorders The 2023 dataset^4^ included responses from 29 113 facilities and had an 84.9% response rate with over 98% of facilities completing the online survey. We omitted hospitals and residential-only treatment facilities, facilities that only serve individuals seeking treatments for driving under the influence or driving while intoxicated; facilities operated by the Department of Veterans Affairs, Department of Defense, or Indian Health Services; and facilities in Puerto Rico or other jurisdictions. Data were based on responses to question, “Which of the following services are provided to clients with co-occurring mental health and substance use at this facility? Mark all that apply.”

## Discussion

Most mental health treatment facilities report the provision of SUD treatment for co-occurring disorders, yet among those that primarily provide mental health treatment, only 10% offered MAUD. Even among facilities that treat both mental health and SUD as primary services, less than half offered MAUD. This suggests patients with co-occurring disorders may receive better access to effective AUD treatment by seeking treatment at facilities that treat both mental health and SUD as primary services.

Reasons for the lack of adoption of AUD treatments are multifactorial. Due to cost or workforce shortages, facilities may lack a prescribing clinician on staff, although we find low adoption of MAUD among facilities that prescribe other medications. Common physician-reported reasons for reluctance to treat SUD include lack of knowledge, institutional environment, and skills.^5^ Addiction specialists may hesitate due to perceived low efficacy, adverse effects, and cost.^6^ The SAMHSA-funded Providers Clinical Support System, which provides training on AUD treatment to health care professionals, may help fill this gap.

Study limitations include that we cannot assess how frequently AUD treatment services are used by patients. Integrated treatment within 1 system is the preferred model, but some individuals may be referred elsewhere for AUD treatment.

## Notes

Supplement.
Data Sharing Statement
