# Supplementary material for: Alcohol Use Disorder Treatment Availability at Mental Health Treatment Facilities
Source: JAMA Netw Open. 2025 Jul 14;8(7):e2520409. doi: 10.1001/jamanetworkopen.2025.20409 (PMC12260999; doi:10.1001/jamanetworkopen.2025.20409)
Supplement: Supplement. — Data Sharing Statement [file jamanetwopen-e2520409-s001.pdf]

Data Sharing Statement

Busch. Alcohol Use Disorder Treatment Availability at Mental Health Treatment Facilities. *JAMA Netw Open*. Published July 14, 2025. doi:10.1001/jamanetworkopen.2025.20409

Data
Data available: No

Additional Information
Explanation for why data not available: The data used in this study is publicly available at https://www.samhsa.gov/data/.
